# Ontology-based collection, representation and analysis of drug-associated neuropathy adverse events

**DOI:** 10.1186/s13326-016-0069-x

**Published:** 2016-05-21

**Authors:** Abra Guo, Rebecca Racz, Junguk Hur, Yu Lin, Zuoshuang Xiang, Lili Zhao, Jordan Rinder, Guoqian Jiang, Qian Zhu, Yongqun He

**Affiliations:** University of Michigan Medical School, Ann Arbor, MI 48109 USA; School of Medicine and Health Sciences, University of North Dakota, Grand Forks, ND 58203 USA; Mayo Clinic, Rochester, MN USA; University of Maryland, Baltimore County, Baltimore, MD 21250 USA; Unit for Laboratory Animal Medicine, Department of Microbiology and Immunology, Center for Computational Medicine and Bioinformatics, and Comprehensive Cancer Center, University of Michigan Medical School, 1301 MSRB III, 1150 W. Medical Dr., Ann Arbor, MI 48109 USA

## Abstract

**Background:**

Neuropathy often occurs following drug treatment such as chemotherapy. Severe instances of neuropathy can result in cessation of life-saving chemotherapy treatment.

**Results:**

To support data representation and analysis of drug-associated neuropathy adverse events (AEs), we developed the Ontology of Drug Neuropathy Adverse Events (ODNAE). ODNAE extends the Ontology of Adverse Events (OAE). Our combinatorial approach identified 215 US FDA-licensed small molecule drugs that induce signs and symptoms of various types of neuropathy. ODNAE imports related drugs from the Drug Ontology (DrON) with their chemical ingredients defined in ChEBI. ODNAE includes 139 drug mechanisms of action from NDF-RT and 186 biological processes represented in the Gene Ontology (GO). In total ODNAE contains 1579 terms. Our analysis of the ODNAE knowledge base shows neuropathy-inducing drugs classified under specific molecular entity groups, especially carbon, pnictogen, chalcogen, and heterocyclic compounds. The carbon drug group includes 127 organic chemical drugs. Thirty nine receptor agonist and antagonist terms were identified, including 4 pairs (31 drugs) of agonists and antagonists that share targets (e.g., adrenergic receptor, dopamine, serotonin, and sex hormone receptor). Many drugs regulate neurological system processes (e.g., negative regulation of dopamine or serotonin uptake). SPARQL scripts were used to query the ODNAE ontology knowledge base.

**Conclusions:**

ODNAE is an effective platform for building a drug-induced neuropathy knowledge base and for analyzing the underlying mechanisms of drug-induced neuropathy. The ODNAE-based methods used in this study can also be extended to the representation and study of other categories of adverse events.

**Electronic supplementary material:**

The online version of this article (doi:10.1186/s13326-016-0069-x) contains supplementary material, which is available to authorized users.

## Background

The word “neuropathy” is derived from two parts: “neuro” referring to the nerve and “pathy” indicating disease. Neuropathy refers herein to nerve damaging. The manifestation of neuropathy often includes chronic pain, loss of sensation, paresthesia, dysesthesia, and motor movement disorders [1]. Drug-induced neuropathies are usually uncommon (2–4 % of cases in one outpatient neurology setting), but crucial to recognize because intervention can lead to significant improvement or symptom resolution [2]. Typically, chemotherapy drugs cause higher incidences of severe neuropathy than other drugs. For example, bortezomib (indicated for multiple myeloma and mantle cell lymphoma) caused treatment-related severe peripheral neuropathy (PN) (grade 3–4) in ~35 % of 331 relapsed multiple myeloma patients (Drugs@FDA). Besides affecting patient quality of life, an effective treatment could be discontinued if PN is intolerable. The signs, symptoms and severity of drug-induced neuropathy are related to many variables such as mechanism of drug action, drug dose, duration of treatment, and host factors. Drug targets in the nervous system are diverse and include cell bodies in the dorsal root ganglia, ion channels, myelin sheath, and neuronal mitochondria. These neurotoxic targets often overlap with drug therapeutic mechanisms. For example, taxanes, which interfere with cell division and apoptosis by binding to β-tubulin subunits, can disrupt axonal transport in neurons and eventually lead to axonopathy. While therapeutic strategies to alleviate neuropathy exist, a better understanding of pathophysiological mechanisms of the drug-induced neurotoxicity is needed to aid the development of novel chemotherapeutics with a lower neurotoxic profile.

The study of drug-associated neuropathy adverse events (AEs) relies on the use of different ontologies. Biomedical ontologies are sets of terms and relations that represent entities in the scientific world and how they relate to each other. Ontologies have been used in applications such as the establishment of knowledge base and computer-assisted automated reasoning. The Ontology of Adverse Events (OAE; http://www.oae-ontology.org/) is a community-based biomedical ontology in the domain of adverse events [3]. OAE provides a logically defined terminology and term relations for various adverse events, including different types of neuropathy adverse events. OAE, together with related theories, also provides a semantic framework that links clinical adverse event phenotypes with underlying biological mechanisms [4, 5]. Drug Ontology (DrON) is a newly generated ontology of drugs and related drug information [6]. DrON incorporates drug information from RxNorm, a normalized drug naming system provided by the National Library of Medicine at NIH [7]. DrON also links drugs to chemical names based on chemical nomenclature as represented in Chemical Entities of Biological Interest (ChEBI) [8]. NDF-RT is another ontology that includes mechanisms of action for drugs. The mechanisms of actions may be linked to Biological Processes, a part of the Gene Ontology (GO) [9]. All these ontologies provide the basis for interdisciplinary study, representation, and analysis of neuropathy adverse events.

By integrating these ontologies with known drug-associated neuropathy AEs, it is possible to generate a domain-specific ontology to represent and study drug-associated neuropathy AEs. In this paper, we report our efforts in developing a community-driven Ontology of Drug Neuropathy Adverse Events (ODNAE). We collected neuropathy-inducing drugs from a number of datasets, ontologically represented the drugs and their mechanisms, and generated scientific insights using ontology-based approaches.

## Methods

### Identification of FDA-approved drugs with neuropathy in their labels

Several methods were applied to identify the US Food and Drug Administration (FDA)-approved drugs known to cause neuropathy. First, our study included a list of neuropathy-associated drugs identified from a previous study using literature mining, survey of three databases (Drugs@FDA, DailyMed, and SIDER), and manual curation [10]. This study uses neuropathy related terms from CTCAE [11] and MedDRA [12]. Secondly, we used an ADEpedia dataset developed at Mayo Clinic (http://adepedia.org) [13] to obtain the information on drugs associated with neuropathy. In the ADEpedia dataset, drugs are represented using the RxNorm codes (i.e., RxCUIs) and AEs are represented using the SNOMED CT [14] codes. Thirdly, we searched LinkedSPLs, a Linked Data resource that published the information of FDA-approved drug package inserts from DailyMed [15]. Lastly, we manually reviewed all the package insert documents and selected drugs after manual confirmation.

### ODNAE editing and existing ontology term import

ODNAE was developed using the format of W3C standard Web Ontology Language (OWL2) (http://www.w3.org/TR/owl-guide/). For efficient editing of OAE, Protégé 4.3 or 5.0 OWL ontology editor (http://protege.stanford.edu/) was used. Based on the annotated data, we used OntoFox (http://ontofox.hegroup.org/) [16] to extract subsets of related terms from different ontologies. Neuropathy AEs from OAE and drugs from DrON, were retrieved and imported to ODNAE, respectively. The mechanisms of most of these drugs are extracted from NDF-RT and imported to ODNAE. Gene Ontology (GO) biological processing terms that match the drug mechanisms were manually identified and imported to ODNAE using OntoFox. Given that many terms from multiple ontologies (OAE, DrON, NDF-RT, and GO) were imported into ODNAE, the alignment of all the imported terms was a challenge and had been solved by a carefully designed strategy to manually assert top level terms of these imported ontology subsets under the ODNAE ontology hierarchical structure. Once the top level terms are aligned, the middle and bottom level ontology terms will be aligned automatically. In addition, we used Ontorat, another internally developed web-based program (http://ontorat.hegroup.org/) [17], to assign RxNorm and NDF-RT identifiers to corresponding DrON drug terms using the annotation property *rdfs:seeAlso*.

### Generation of new ODNAE terms and axioms related to drug-induced neuropathy AEs

Ontorat was used to generate specific drug-induced neuropathy AE terms with “ODNAE_” prefix, and define new axioms to link the newly generated ODNAE terms with corresponding drugs and neuropathy AEs. To run the Ontorat program, all the related data were formalized into a structure Excel template. Ontorat scripts were developed to identify sets of data and insert them into ODNAE under appropriate hierarchical structures.

### ODNAE access, visualization, and licensing

The ODNAE project website is located at Github: https://github.com/odnae. ODNAE has been deposited in the repositories of Ontobee (http://www.ontobee.org/ontology/ODNAE) and NCBO BioPortal (http://bioportal.bioontology.org/ontologies/ODNAE). The ODNAE source code is also freely available under the Creative Commons 3.0 License (http://creativecommons.org/licenses/by/3.0/). This licensing allows ODNAE users to freely distribute and use ODNAE.

### SPARQL query of ODNAE

The Ontobee [18] SPARQL query web page (http://www.ontobee.org/sparql) was used to perform SPARQL queries of the ODNAE ontology to answer specifically designed questions. In total, six files of 20 SPARQL scripts were generated for this study. These files are stored on the Github website: https://github.com/odnae/odnae/tree/master/docs/SPARQL. Additional file 1 contains a summary of these 20 scripts.

### Heatmap analysis of ODNAE data

The correlation between drug molecular entities and adverse events were presented using a heatmap. The heatmap was created using n × m count matrix, where n is the number of AEs and m is the number of drug molecular entities (i.e., the top level drug chemical entity groups). Each cell in the matrix is the number of drugs under the drug chemical group (i.e., column) that are associated with a specific AE (i.e., the row). The matrix was generated by first using SPARQL to obtain the raw data of each drug chemical group, drug, and drug-associated AEs, and using an R program to process and transfer the data to the desired format. The heatmap was ordered using the Manhattan distance and the molecular entities were clustered using the complete linkage method. The heatmap was plotted using R 3.1.3.

## Results

The overall goal of this project is to generate and analyze an ontology knowledge base of drug-associated neuropathy AEs. By “ontology knowledge base”, we mean that the ontology itself serves as a knowledge base that integrates various aspects of knowledge related to a specific domain, promoting knowledge integration and discovery. Therefore, the ODNAE serves as a knowledge base comprising drug components, chemical entities of active drug ingredients, drug mechanisms, and drug-inducing neuropathy AEs. To achieve this goal, we first used different methods to identify drugs associated with different types of neuropathy AEs. Related information was then represented in the ODNAE and further analyzed.

In what follows, single quotation marks ‘’ are used to quote specific ontology terms.

### Drugs associated with neuropathy adverse events

As described in the Methods section, three methods (i.e., literature mining followed by manual curation [10], ADEpedia query [13], and LinkedSPLs query [15]) were used to obtain drugs associated with neuropathy AEs. Each of these methods identified from 150-230 neuropathy-inducing drugs. After a second round of manual verification, we verified 215 chemical drugs known to induce neuropathy AEs. This list of drugs does not include 36 drugs that were originally identified from our data sources due to either a lack of DrON IDs, a clear label of a neuropathy AE, or absence of a subclass of neuropathy AE. It is noted that the data from user-reported FDA adverse event case reporting systems (FAERS) [19] were not used since the FAERS results can be quite noisy. An Excel file containing 215 annotated drugs and neuropathy AEs is stored in the ODNAE GitHub repository: https://github.com/odnae/odnae/raw/master/src/ontology/Ontorat_inputs/odnae-data-outputupdate.xlsx.

### General ODNAE design and statistics

**Fig. 1:**
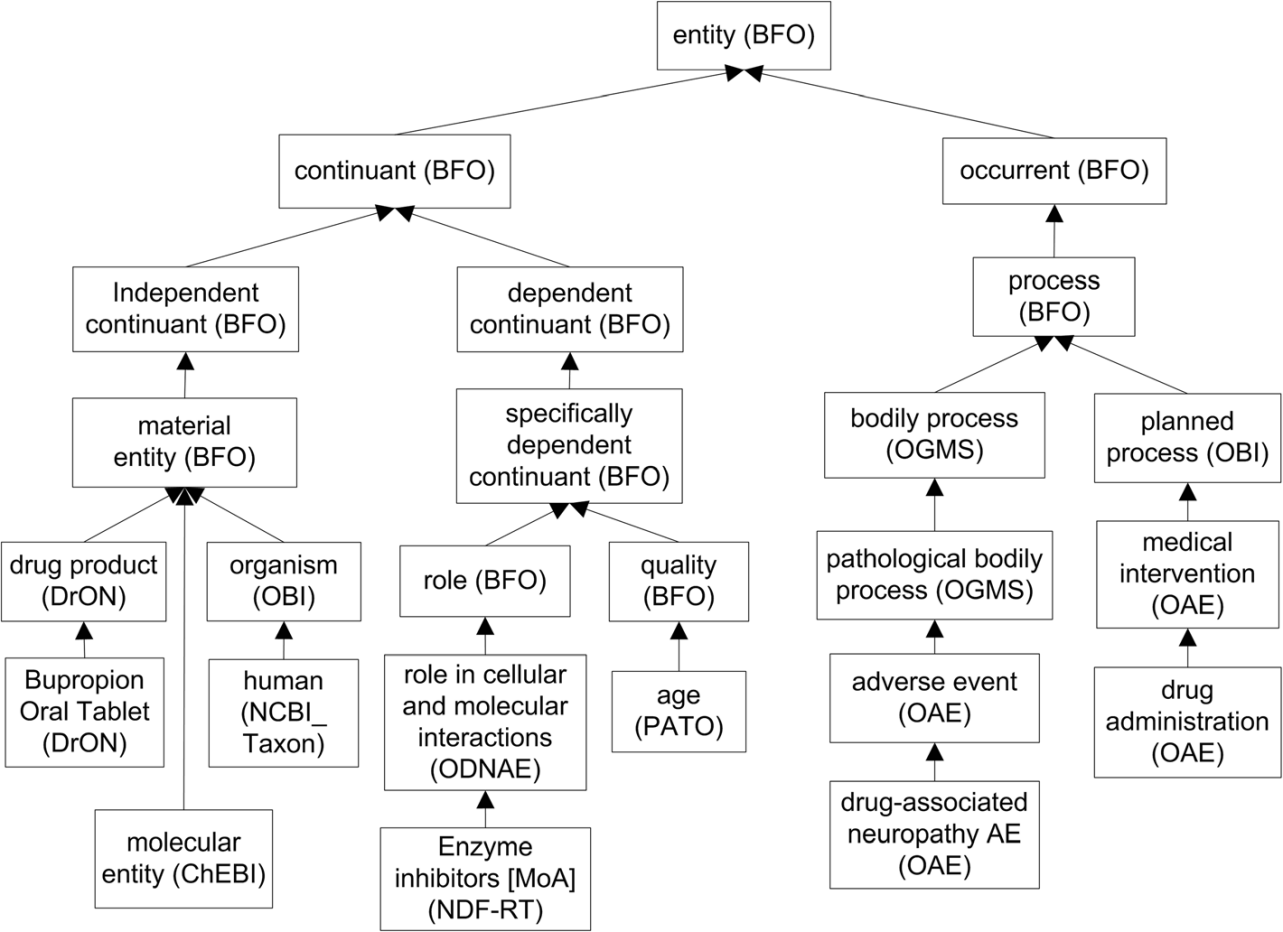
Top level ODNAE hierarchy

The top level hierarchy of ODNAE is demonstrated in Fig. 1 and explained below.

First, ODNAE extends OAE and reuses the upper level of OAE. Like OAE, ODNAE uses the Basic Formal Ontology (BFO) [20] as the upper level ontology. BFO contains two branches, ‘continuant’ and ‘occurrent’ [21, 22]. The ‘continuant’ branch represents time-independent entities such as material entity and quality. The ‘occurrent’ branch represents time-related entities such as adverse event, drug administration, drug metabolism, and dose accumulation in human. By aligning different terms under the two branches of BFO, knowledge from broad biological areas related to drug-associated neuropathy AEs were captured and organized under a unified ontology-level structure.

Among several drug ontologies (RxNorm, NDF-RT, and DrON), we selected DrON as the default ontology for representing drugs, as DrON allows mapping between drugs and ChEBI chemical terms. In addition, like ODNAE and OAE, DrON is also aligned with BFO. The advantage of using BFO is that BFO has been adopted by over 100 biomedical ontologies. All these ontologies follow ontology design principles of the Open Biomedical Ontologies (OBO) Foundry [22]. Therefore, we were able to easily import and integrate related terms from DrON, OAE, and other OBO ontologies into ODNAE. In order to enable data integration and data reuse, we added links from the DrON terms to RxNorm and NDF-RT IDs by annotation property *rdfs:seeAlso* in ODNAE.

**Fig. 2:**
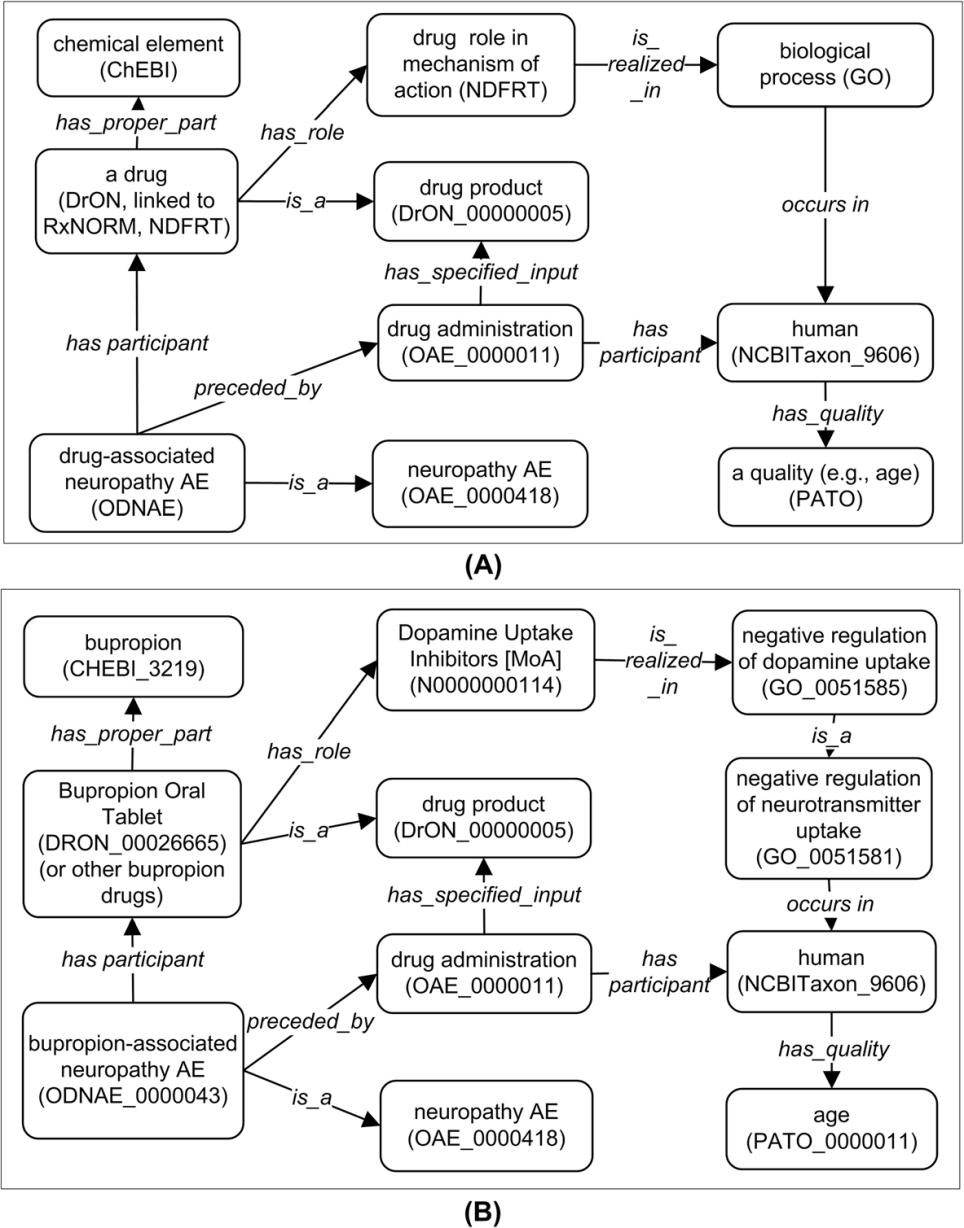
ODNAE design pattern and example. **a** ODNAE design pattern of representing drug-associated neuropathy AE. **b** ODNAE representing bupropion-associated neuropathy AE

Figure 2 shows the basic design pattern of ODNAE representation of drug-associated neuropathy AEs (Fig. 2a) and one example of implementing the design (Fig. 2b). Specifically, a ‘drug-associated neuropathy AE’ (e.g., ‘bupropion-associated neuropathy AE’) occurs after (‘preceded_by’) an administration of a drug (e.g., Bupropion Oral Tablet or Aplezin) in a ‘human’ patient. The human has different qualities (such as ‘age’, ‘gender’, and ‘disease history’) and genomics background which may affect adverse event outcomes. The drug has a proper component of a molecular entity (e.g., bupropion). The drug also has a specific role in a biological process. The NDF-RT mechanism of action (MoA) terms (e.g., dopamine uptake inhibitor) is represented as ‘role’ (BFO_0000023), which is realized in a Gene Ontology (GO) biological process (e.g., ‘negative regulation of dopamine uptake’ GO_0051585) (Fig. 2).

The linked information illustrated in Fig. 2 is logically defined in ODNAE. Logical constraints allow proper integration and hierarchies among terms from different ontologies of drugs, the chemicals of active drug ingredients, GO processes, and other information cross-linked with axioms. As a result, the ontology knowledge base of drug neuropathy AEs can be analyzed at different levels of classification.

As shown in Figs. 1 and 2, ODNAE imports terms from many existing ontologies and also contains newly generated, ODNAE-specific terms. In total, ODNAE contains 1579 terms, including 249 terms with “ODNAE_” prefix and terms imported from other ontologies such as 25 OAE terms, 500 ChEBI terms, and 331 DrON terms. The detailed statistics of ODNAE is available at the Ontobee website: http://www.ontobee.org/ontostat/ODNAE.

In the next sections, we will provide more details about the ODNAE contents and scientific insights from ODNAE data analysis.

### Various types of neuropathy AEs are associated with drugs

**Fig. 3:**
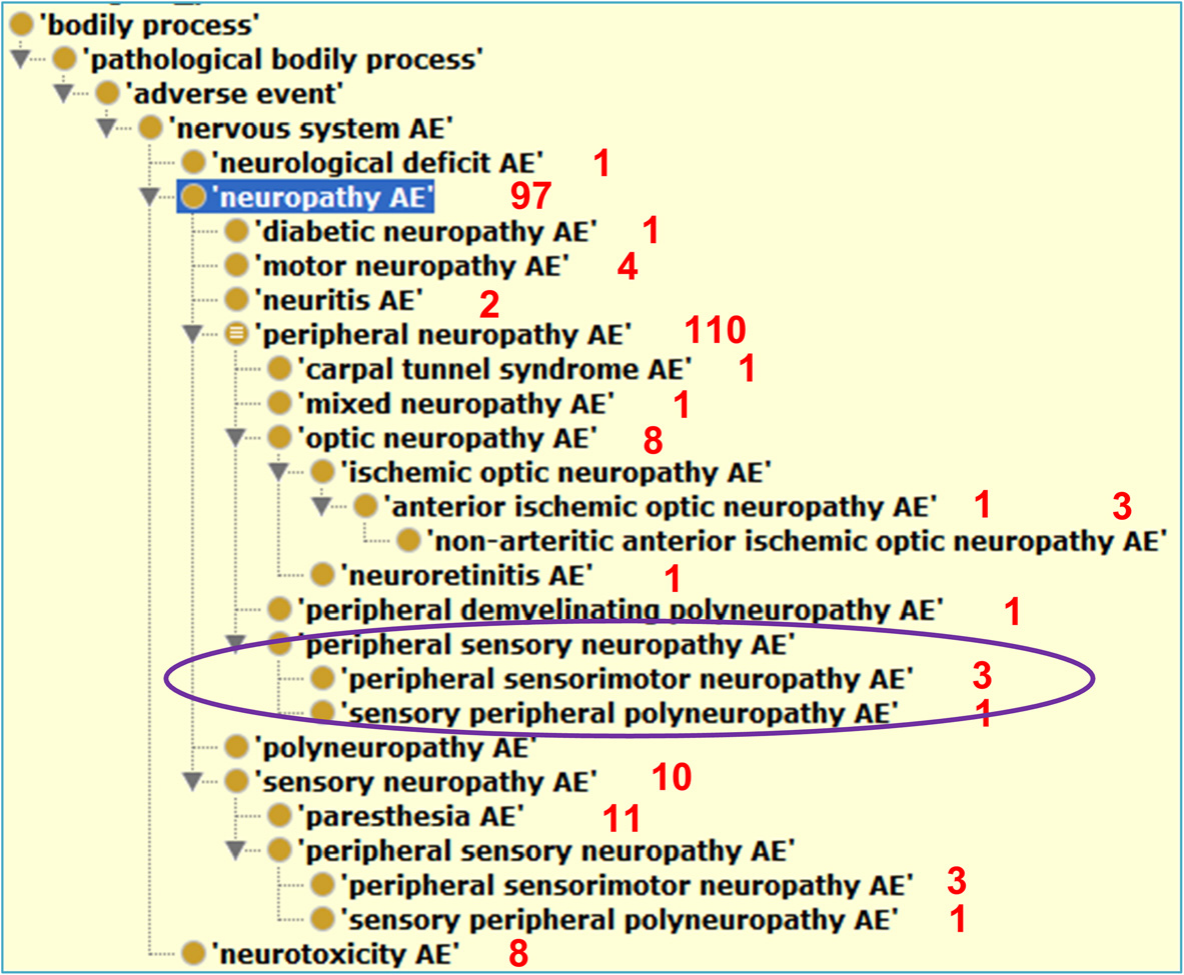
Various drug-associated neuropathy AEs as represented in OAE and imported to ODNAE. *Red numbers* represent the numbers of drugs associated with the corresponding AEs. *Circled* are 3 terms not asserted but inferred under ‘peripheral neuropathy AE’ using a Hermit reasoner in Protege OWL editor

Our study identified 20 types of neuropathy AEs, each of which is associated with at least one drug (Fig. 3). Represented in a hierarchical structure, these AEs are logically defined and cross-referenced to existing AE representation systems including MedDRA [12].

### Ontology representation of neuropathy-associated drugs and drug ingredients

The active ingredient of a drug product plays a vital role in its mechanism. The chemical structures of the drug active ingredients are represented in ODNAE using ChEBI terms. Additional ChEBI terms are also imported to form the hierarchy of these active ingredients of drugs. The relation between a drug and a ChEBI chemical is presented by an object property ‘has_proper_part’ (Fig. 2).

**Fig. 4:**
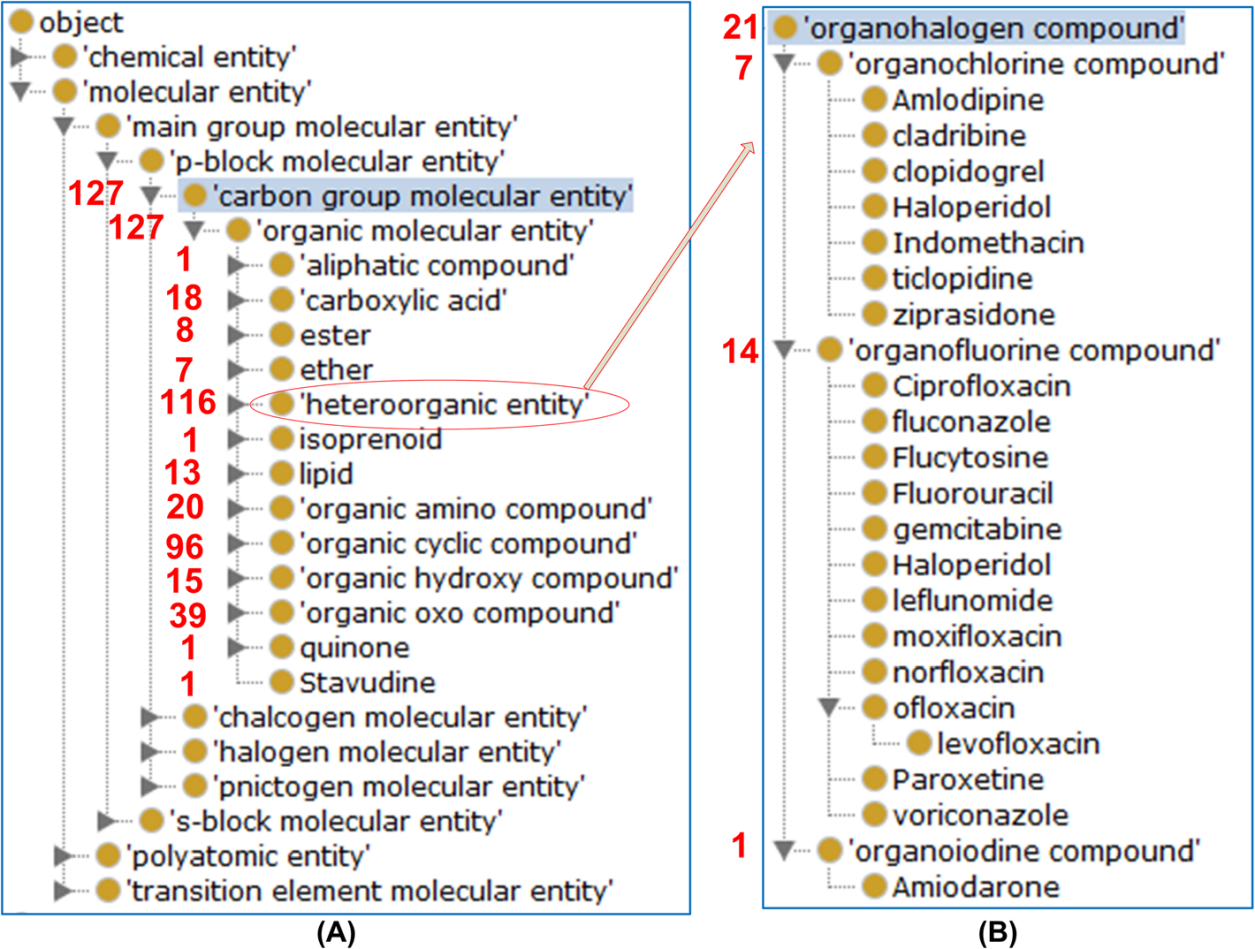
Example ChEBI classification of drug chemicals inducing neuropathy AEs. **a** 14 neuropathy-inducing drugs are classified under nucleotide. **b** 21 drugs containing organohalogen compounds as active ingredients were found to induce neuropathy AEs

Most drug-associating ChEBI terms are under the branch of ‘molecular entity’ (CHEBI_23367). There are 23 classes, for example, ‘carbon group molecular entity’ (CHEBI_33582), at the third layer below ChEBI term ‘molecular entity’ (Fig. 4a). Among all these 23 classes, the ‘carbon group molecular entity’ class is associated with 127 drugs (the highest number). All drugs under this group were indeed all organic molecular entities (Fig. 4a). Among 13 subclasses of organic entities, heterorganic entities link to 116 neuropathy-inducing drugs (Fig. 4a). Figure 4b provides an example of a subclass of heterorganic entities (Fig. 4b). All the results can be counted from the ontology display in the Protégé OWL editor. Alternatively, as detailed later, a SPARQL script can obtain the same count results.

#### Ontology-based representation and analysis of drug mechanisms

A total of 139 mechanisms of action (MoA) terms related to neuropathy-inducing drugs was identified from NDF-RT and imported to ODNAE. We identified 13 GO biological processes that directly realize roles, or MoAs from NDF-RT. Many MoA terms do not have matched GO terms. ODNAE also includes 173 GO terms that are the ancestor (or related) terms of these 13 GO terms.

**Fig. 5:**
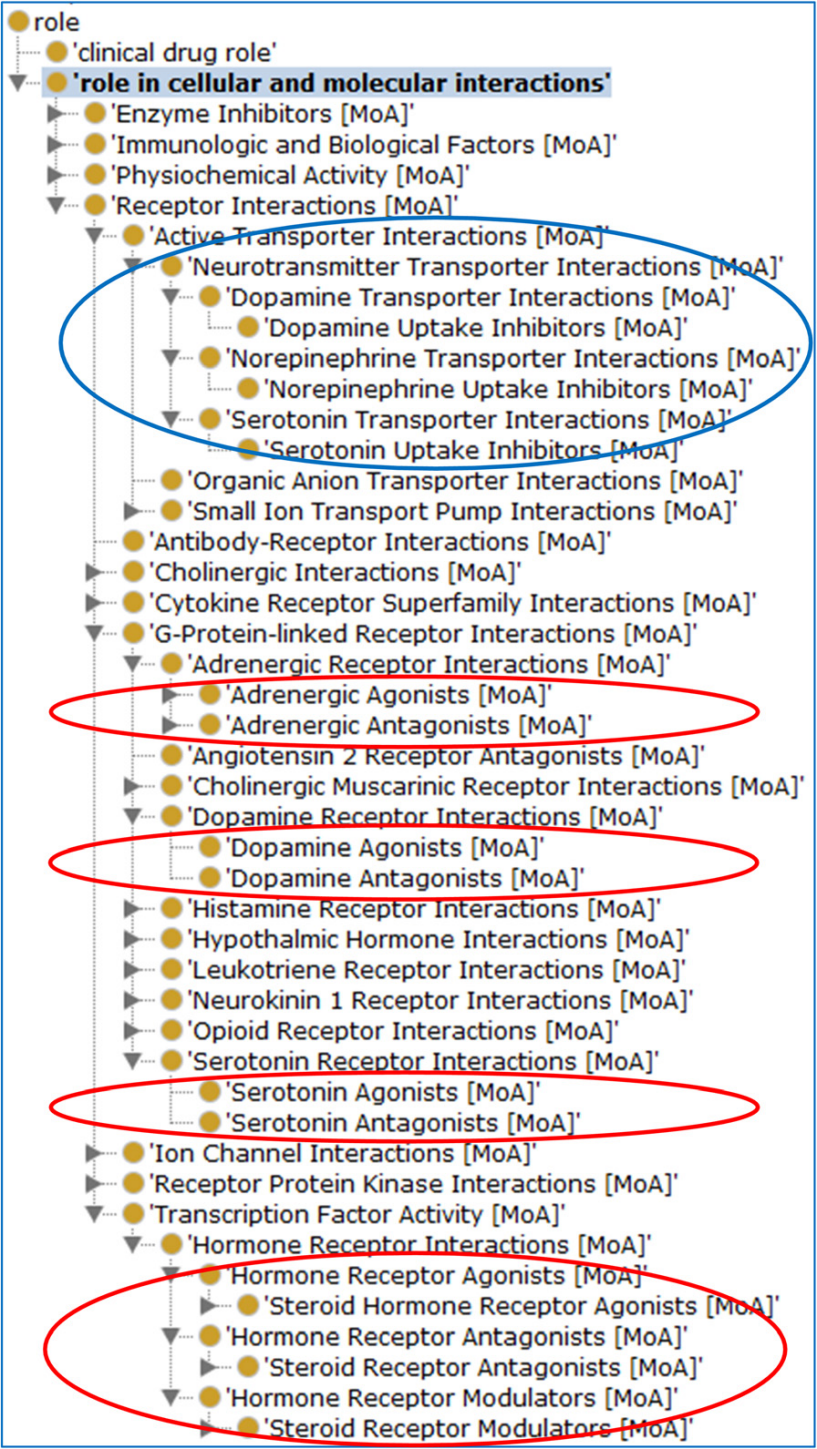
Various roles in cellular and molecular interactions played by drugs associated with neuropathy AEs. The branch *circled in blue* indicates inhibitor roles related to neurotransmission. Roles *circled in red* indicate either agonists or antagonists associated with neuropathy AEs

Much insight was gained by examining the NDF-RT MoAs collected in ODNAE (Fig. 5). All NDF-RT roles were organized as subclasses of ‘role in cellular and molecular interactions’, including the roles as ‘enzyme inhibitors’, ‘immunological and biological factors’, and receptors of different biological interactions. Our results showed that 12 neuropathy AE related drugs inhibit the uptake of three neurotransmitters [dopamine (1), norepinephrine (10), and serotonin (11)]. There are 20 drugs that interact with the G-protein receptors that contribute to neuropathy adverse events. We identified 39 drug agonist and antagonist terms, including 16 agonists and 23 antagonists. Among them, there are four pairs of agonists and antagonists of the same targets (Table 1). Specifically, there are 3 agonist drugs and 7 antagonist drugs of the adrenergic receptor, 4 agonists and 2 antagonists of dopamine, 4 agonists and 3 antagonists of serotonin, and 5 agonists and 2 antagonists of hormone receptor (Table 1). In addition, there are 3 hormone receptor modulators (i.e., Leuprolide, Leuprolide acetate, and Taxoxifen) that are also associated with neuropathy AE (Table 1).

**Table 1:** Four pairs of agonists and antagonists of neuropathy-inducing drugs

Target	Agonists/antagonists	Drugs
adrenergic receptor	agonists	3: Tizanidine, Salmeterol, Salmeterol xinafoate
	antagonists	7: Ziprasidone, Amiodarone, Amiodarone HCL, Propafenone, Betaxolol, Sotalol, Sotalol HCL
dopamine	agonists	4: Bromocriptine, Pergolide, Pramipexole, Ropinirole
	antagonists	2: Haloperidol, Ziprasidone
serotonin	agonists	4: Almotriptan, Eletriptan, Sumatriptan, Zolmitriptan
	antagonists	3: Alosetron, Cyproheptadine, Ziprasidone
hormone receptor	agonists	5: Nevirapine, Megestrol, Dexamethasone, Fluticasone, Fluticasone propionate,
	antagonists	2: Bicalutamide, Megestrol
	modulators	3. Leuprolide, Leuprolide acetate, Taxoxifen

The GO terms in ODNAE cover a variety of processes, including negative regulation of neurotransmitter uptake and synaptic transmission. GO terms are linked to genes and proteins. We will investigate in the future how ODNAE can represent gene/protein-based neuropathy mechanisms with the support of GO.

#### Query of drug-induced neuropathy AEs

**Fig. 6:**
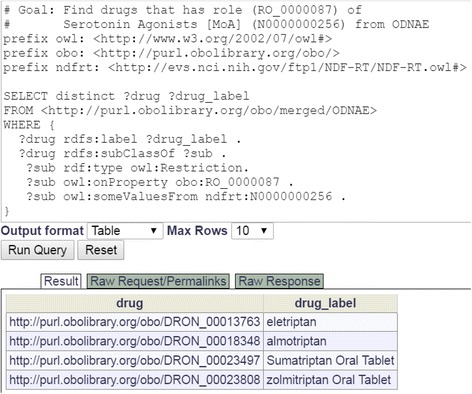
SPARQL query of drugs acting as a serotonin agonist. The query was done in Ontobee SPARQL website (http://www.ontobee.org/sparql)

The ODNAE knowledge base can be queried through the Ontobee SPARQL program. Different questions can be addressed using SPARQL queries. For example, a SPARQL script was generated to identify what drugs act as a serotonin agonist (Fig. 6). The query resulted in four drugs: eletriptan, almotriptan, sumatriptan, and zolmitriptan.

In addition to the query shown in Fig. 6, many other SPARQL scripts were also generated to meet different requirements for many studies introduced in this article, we have generated many SPARQL scripts. All these query scripts have been collected and provided in the Additional file 1.

#### Heatmap analysis of the correlations between drug molecular entities and neuropathy AEs

**Fig. 7:**
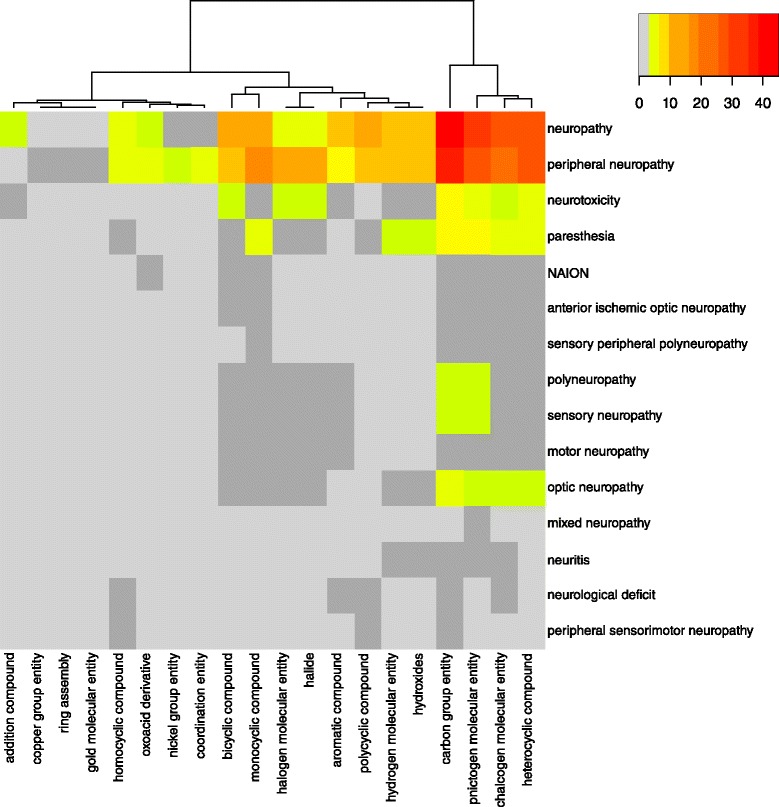
Heatmap analysis of drug molecular entity-AE relations. Drug molecular entities include 20 DrON terms at the third layer under ChEBI term ‘molecular entity’. Color scheme indicates the numbers of AEs for different groups of drugs: *light grey* is 0, *dark grey* is 1, and the rest are ordered by *yellow*, *orange* and *red*

One question is how to correlate the drug molecular entity group with specific neuropathy AEs. To address this question, a heatmap analysis was performed (Fig. 7). The data for the heatmap analysis was achieved using SPARQL in the Methods section. The data obtained from SPARQL queries include the drug molecular entities and AEs that are associated with different drugs in ODNAE. The heatmap explores the relation between drug molecular entities and various neuropathy AEs (Fig. 7).

Our results showed that drug-associated ‘carbon group molecular entities’ (CHEBI_33582), pnictogen (CHEBI_33302), chalcogen compounds (CHEBI_33304), and heterocyclic compounds (CHEBI_5686) were associated with the highest numbers of AE cases, and these four groups of chemicals also form a cluster by themselves in the heatmap analysis (Fig. 7). The chemicals in each group are also associated with different types of neuropathy AEs. For example, in the carbon group molecular entities, 45 drugs are directly associated with the top level neuropathy AE, 40 drugs associated with peripheral neuropathy AE, 6 drugs with neurotoxicity AE, and 5 with paresthesia AE. In addition to the four groups of chemicals with the highest numbers of neuropathy-inducing drugs, other groups of chemicals, including monocyclic, bicyclic, and polycyclic compounds, are also associated with high numbers of neuropathy-inducing drugs. The chemical groups that are the least associated with neuropathy AEs include copper group entity, ring assembly, and gold molecular entity chemicals (Fig. 7).

## Discussion

The contributions of this article are multiple. First, 215 neuropathy-inducing drugs were manually collected and curated from different reliable resources. Second, ODNAE serves as a ontology knowledge base that represents drug-induced neuropathy AEs and links these AEs to different sets of entities (e.g., drugs, chemical characteristics, drug targets, drug mechanisms of action, and biological processes). Third, the knowledge in the ODNAE knowledge base was analyzed for obtaining scientific insights into drug-associated neuropathy AEs from different aspects, including related neuropathy AE classifications, chemical structure patterns, findings from mechanisms of actions, and the heatmap relations between chemical classifications and AE types. ODNAE also provides a semantic platform for further knowledge addition/integration and advanced analysis. For example, the ODNAE framework can be extended for other drug and AE studies.

Different from many reference ontologies (e.g., OAE and DrON) that represent terms and relations among the terms in a specific domain (e.g., adverse events and drugs), ODNAE serves as a ontology knowledge base that reuses reference ontology terms and provides logical axioms to link different pieces of information such as neurophathy AEs, drugs, chemical elements of drug active ingredients, and mechanisms of action. As a knowledge base, ODNAE captures knowledge extracted from biomedical bench research, clinical practices, and public health. Owing to the parsable and machine readable nature of the AE knowledge base, ODNAE supports neuropathy AE data exchange, data integration, and automated reasoning and classification.

To demonstrate the advantages of ontology-supported data integration and classification, we have mined the ODNAE knowledge base through systematic classification and statistical analysis and obtained many scientific findings from this study. First, we identified the neuropathy AE types induced by 215 drugs. Our systematic classification identified the major drug chemical element groups (e.g., carbon molecular groups) and their subgroups that are associated with neuropathy AEs (Figs. 4 and 7). We have also found an interesting observation that many agonists and antagonists of the same targets (e.g., dopamine, serotonin, and sex hormone receptor) both lead to neuropathy AEs (Fig. 5 and Table 1). Such observation suggests that these target molecules require a balanced level in the host, and too high or too low may lead to neuropathy AEs. We have also generated a heatmap to further identify the relations between drug chemical entities and different types of neuropathy AEs (Fig. 7).

It is noted that many findings from our ontology knowledge base analysis have been reported in the literature [23–31]. For example, agonists and antagonists of the same targets associated with neuropathy AEs have been reported previously [23, 24]. Specific chemical structures, which are among the structures found in our analysis, have been found to be required for the induction of neuropathy [25–31]. These literature reports indeed confirm our analysis results from the usage of the ontology-based neuropathy-inducing drugs as the only input data. Given the complete list of the neuropathy-inducing drugs in our study, our analysis also provides a comprehensive view of features covered in the ontology. In addition, our ontology-based strategy generates a semantic framework that brings related information together in a structured and logical format and supports knowledge integration and analysis. Such a machine-readable ODNAE framework is novel and has not been reported in any neuropathy adverse event studies. ODNAE also provides a basis for educational learning, further extension, and interaction with external domains of knowledge to support integrative neuropathy pharmacovigilance research.

Beyond the paper’s primary focuses on the data collection, ontology representation, and ONDAE data analysis for discovering scientific insights, ODNAE can be further used for more case studies in the future. For example, the integrated ODNAE knowledge and data can be used to possibly predict potential neuropathy AEs for particular drugs based on the structures of the drugs that have been enriched in our study. Our study found that over half of the neuropathy-inducing drugs are organic carbon molecules with special enrichment on heteroorganic and organic cyclic compounds (Fig. 4). It is known that some specific chemical structures are required for the induction of neuropathy [25–31]. For example, 1,2-diacetylbenzene (1,2-DAB) (but not its isomer 1,3-DAB), 1,2-Diethylbenzene (1,2-DEB), and 1,2,4-Triethylbenzene (1,2,4-TEB) are able to induce chromogenic changes and neurotoxicity; and the 1,2-spaced ethyl (or acetyl) moieties on a benzene ring of these hydrocarbons have been found to be a critical molecular arrangement resulting neurotoxic properties [25–27]. It is interesting that our results show 3 benzene drug compounds (i.e., fentanyl, sulfasalazine, and acetylsalicylic acid) and 3 other benzoid drug compounds (i.e., mitoxantrone, fluoxetine, and losartan) are also associated with neuropathy AEs. Any specific structures in these and other drug chemical compounds that may facilitate neuropathy processed require further analysis. It is likely that structural similarity analysis combined with biological studies [28–31] could be conducted among these drugs. To validate the association between drug structures and specific neuropathy, observational clinical trials and laboratory experiments with valid animal models can be considered. If a structure (e.g., 1,2-spaced ethyl moieties on a benzene ring) is found to be more preferentially than others in inducing neuropathy, we can specifically avoid or modify the structure (with a balance of efficiency) to increase drug safety.

Another future use of ODNAE is to make ODNAE a platform to model and represent other information related to neuropathy-inducing drugs. Chemical characteristics of drug, drug disposition in humans, and patient factors could all play a role in the induction of adverse drug events. For example, drug dosage, environmental factors, individual patient age, disease, genotype (e.g., genetic variations compared to others), and physiological conditions each plays a critical role in specific drug neuropathy AEs. These parameters can be linked to other elements presented in the ODNAE semantic framework. Such an ontology-based semantic framework can also be guided by related biological network theories, including the OneNet Theory of Life [5, 32]. The ODNAE-based and theory-guided integrative analysis would be able to identify relations between those factors and drug-associated neuropathy. Therefore, our work defines a very important framework for understanding drug-induced peripheral neuropathy. Ultimately it will allow us to advance personalized medicine, including the development of neuroprotective strategies for cancer patients or patients suffering from neurological disorders such as diabetic neuropathy.

Our ODNAE will be continuously expanded and computerized to integrate multiple layers of information, including chemical characteristics of drugs, biological receptors and processes at the cellular and molecular levels, drug disposition in patients, pharmacogenetics, and population level variables. Drug-associated neuropathy AEs are most likely associated with various personal backgrounds such as age, gender, and genotype. ODNAE can be expanded to cover these more personalized factors to find trends in neuropathy and better predict events.

## Conclusions

Drugs of diverse pharmacological classes may cause different levels of neuropathy AEs. In this study, 215 drugs were collected and represented in the Ontology of Drug Neuropathy Adverse Events (ODNAE). ODNAE serves as a knowledge base that reuses existing ontologies and includes logical axioms to represent the relations among different entities including these 215 drugs, drug-associated chemical elements, specific neuropathy types, mechanisms of drug action, and biological processes. The analyses of logically formed ODNAE information revealed remarkable scientific insights into drug-associated neuropathy adverse events. Particularly, our study found different types of neuropathy AEs induced by these 215 drugs, major neuropathy-inducing drug chemical entity groups, the observation of agonists and antagonists of the same targets that are associated with neuropathy AEs, and specific relations between chemical groups and types of neuropathy AEs. These findings are consistent with existing reports, further confirming the validity of our ontology-based analyses that use the list of neuropathy-inducing drugs as the only input. Overall, ODNAE provides a useful platform for integrating and analyzing currently known information related to drug-induced neuropathy AEs and is extensible for future new knowledge representation, analysis and discovery.

## Additional file

Additional file 1: SPARQL scripts developed for the ODNAE analyses used in the ODNAE manuscript. (PDF 475 kb)

## Abbreviations

AE: adverse event
CTCAE: common terminology criteria for adverse events
DL query: description logics query
FDA: Food and Drug Administration
NCBO: The National Center for Biomedical Ontology
OAE: ontology of adverse events
OBI: ontology for biomedical investigations
OBO: The Open Biological and Biomedical Ontologies
ODNAE: ontology of drug neuropathy adverse events
OWL: web ontology language
RDF: resource description framework
SPARQL: SPARQL protocol and RDF query language
