# Supplementary material for: Ontology-based collection, representation and analysis of drug-associated neuropathy adverse events
Source: J Biomed Semantics. 2016 May 21;7:29. doi: 10.1186/s13326-016-0069-x (PMC4875649; doi:10.1186/s13326-016-0069-x)
Supplement: Additional file 1: — SPARQL scripts developed for the ODNAE analyses used in the ODNAE manuscript. (PDF 475 kb) [file 13326_2016_69_MOESM1_ESM.pdf]

# Supplemental File 1:

**SPARQL scripts developed for the ODNAE analyses used in the ODNAE manuscript:**

Note: Six sets of SPARQL scripts (20 individual scripts in total) were generated for the ODNAE study. All the four sets of SPARQL scripts were stored in the ODNAE Github folder: https://github.com/odnae/odnae/tree/master/docs/SPARQL

The contents of these six sets of 20 SPARQL scripts are provided below:

The content of SPARQL File 1:
Title: **query_G_protein_linked_receptor_interaction_drugs**
**Script:**

- **1.1.** SPARQL query for searching the ODNAE drugs annotated with G-Protein-linked Receptor Interactions [MoA] (N0000000152) and its subclasses.

The content of SPARQL File 2:
Title: **query_agonists_ antagonists**

- **2.1.** drugs link to agonist
- **2.2**. The agnoist that links to ODNAE drugs
- **2.3**. drugs and agonist pair:
- **2.4**. drugs link to antagonist
- **2.5**.The antagnoist that links to ODNAE drugs
- **2.6**. Antagonist and drugs pair
- **2.7**. drug link to both a kind of agonist and antagonist
- **2.8.** query drugs that have a specific role, e.g., Serotonin Agonists [MoA]. (used for Fig. 6)

The content of SPARQL File 3:
Title: **query_all_drug_with_MoAs**

- **3.1.** SPARQL query for searching all ODNAE drugs link to MoAs : subClassOf http://purl.obolibrary.org/obo/ODNAE_0000002 (role in cellular and molecular interactions)

The content of SPARQL File 4:
Title: **query_neurotransmitters_interaction_drugs**

- **4.1.** SPARQL query for searching the ODNAE drugs annotated with Neurotransmitter Transporter Interactions [MoA] N0000000105) and its subclasses.
- **4.2.** # SPARQL query for searching the ODNAE drugs annotated with Dopamine Transporter Interactions [MoA] N0000000219) and its subclasses.
- **4.3.** SPARQL query for searching the ODNAE drugs annotated with Norepinephrine Transporter Interactions [MoA] (N0000000234) and its subclasses.
- **4.4.** SPARQL query for searching the ODNAE drugs annotated with Serotonin Transporter Interactions [MoA] (N0000000167) and its subclasses.

The content of SPARQL File 5: (Note: The file to be generated)
Title: **link_chemicals_and_drugs**

- **5.1.** identify the drugs having proper part of chemical elements

- **5.2.** identify all ancestor CHEBI terms of CHEBI_9667 (Triamcinolone)
- **5.3**: get drugs for all child levels of a parent CHEBI term
- **5.4.** Goal: get drugs for all levels of chebi terms --> #2 cleanup

SPARQL File 6: (Note: The file to  be generated)
Title: **link_drugs_Chebi_AEs**

- **6.1**: This is to find drugs vs AEs that are associated with these drugs.
- **6.2**: This is to find AE and related drugs for the AE.
- **6.3:** This is to identify chebi chemical terms for a specific AE. (Note:  This is the script used to generate data for heatmap.)

## The Content of SPARQL file 1:

**Script file name: query_G_protein_linked_receptor_interaction_drugs**
Content:
--

```
# SPARQL 1.1
# SPARQL query for searching the ODNAE drugs annotated with G-Protein-linked Receptor Interactions
[MoA] (<http://evs.nci.nih.gov/ftp1/NDF-RT/NDF-RT.owl#N0000000152>) and its subclasses.

prefix rdf: <http://www.w3.org/1999/02/22-rdf-syntax-ns#>
prefix owl: <http://www.w3.org/2002/07/owl#>
prefix obo: <http://purl.obolibrary.org/obo/>
SELECT distinct ?s ?label
From <http://purl.obolibrary.org/obo/merged/ODNAE>
WHERE
{ ?s rdfs:subClassOf ?s1 .
  ?s rdfs:label ?label .
  ?s1 owl:onProperty obo:RO_0000087; owl:someValuesFrom ?s2 .
  ?s2 rdfs:subClassOf <http://evs.nci.nih.gov/ftp1/NDF-RT/NDF-RT.owl#N0000000152>  option
(transitive).
}
```

***Notes***:
*To count the total number (instead of providing the list):*

- Change “distinct ?s ?label” TO: “count(distinct ?s)” in the script.

*Related terms used in the script:*

- http://purl.obolibrary.org/obo/RO_0000087 --> has role
- ODNAE: N0000000152: http://evs.nci.nih.gov/ftp1/NDF-RT/NDF-RT.owl#N0000000152 : http://www.ontobee.org/ontology/ODNAE?iri=http://evs.nci.nih.gov/ftp1/NDF-RT/NDF-RT.owl%23N0000000152 → G-Protein-linked Receptor Interactions [MoA]

# results: (run on http://sparql.hegroup.org/sparql/, at 8:40 CST 4/30/2015 by Asiyah Yu Lin)

20

s        label

http://purl.obolibrary.org/obo/DRON_00020516 Amantadine Oral Tablet
http://purl.obolibrary.org/obo/DRON_00022309 Losartan Oral Tablet
……
http://purl.obolibrary.org/obo/DRON_00020955 Betaxolol Oral Tablet
--

## The Content of SPARQL File 2:

Script file name: **query_agonists_ antagonists**
Content:
--
# SPARQL query for searching the ODNAE drugs are agonists and antagonists

**# SPARQL 2.1.** drugs link to agonist

```
prefix rdf: <http://www.w3.org/1999/02/22-rdf-syntax-ns#>
prefix owl: <http://www.w3.org/2002/07/owl#>
prefix obo: <http://purl.obolibrary.org/obo/>
SELECT distinct ?s ?label
From <http://purl.obolibrary.org/obo/merged/ODNAE>
WHERE
{ ?s rdfs:subClassOf ?s1 .
  ?s rdfs:label ?label .
  ?s1 owl:onProperty obo:RO_0000087; owl:someValuesFrom ?s2 .
  ?s2 rdfs:subClassOf <http://purl.obolibrary.org/obo/ODNAE_0000002> option (transitive).
  ?s2 rdfs:label ?label2 .
  FILTER REGEX(str(?label2), "Agonist")
}
```

# results in total 17 drugs: (run on http://sparql.hegroup.org/sparql/, at 9:56 CST 4/30/2015 by Asiyah Yu Lin)

s label
http://purl.obolibrary.org/obo/DRON_00013678 cevimeline
http://purl.obolibrary.org/obo/DRON_00022889 Pergolide Oral Tablet
……
http://purl.obolibrary.org/obo/DRON_00022385 Megestrol Oral Suspension

**# SPARQL 2.2**. The agnoist that links to ODNAE drugs

```
prefix rdf: <http://www.w3.org/1999/02/22-rdf-syntax-ns#>
prefix owl: <http://www.w3.org/2002/07/owl#>
prefix obo: <http://purl.obolibrary.org/obo/>
SELECT distinct ?s2 ?label2
From <http://purl.obolibrary.org/obo/merged/ODNAE>
WHERE
{ ?s rdfs:subClassOf ?s1 .
```

```
  ?s rdfs:label ?label .
  ?s1 owl:onProperty obo:RO_0000087; owl:someValuesFrom ?s2 .
  ?s2 rdfs:subClassOf <http://purl.obolibrary.org/obo/ODNAE_0000002>  option (transitive).
  ?s2 rdfs:label ?label2 .
  FILTER REGEX(str(?label2), "Agonist")
}
```

# results in total 9 agonists: (run on http://sparql.hegroup.org/sparql/, at 9:14 CST 4/30/2015 by Asiyah Yu Lin)

s2 label2
http://evs.nci.nih.gov/ftp1/NDF-RT/NDF-RT.owl#N0000000104 Cholinergic Muscarinic Agonists [MoA]
http://evs.nci.nih.gov/ftp1/NDF-RT/NDF-RT.owl#N0000000117 Dopamine Agonists [MoA]
……
http://evs.nci.nih.gov/ftp1/NDF-RT/NDF-RT.owl#N0000000185 Progestational Hormone Receptor Agonists [MoA]

**# SPARQL 2.3**. drugs and agonist pair:

```
prefix rdf: <http://www.w3.org/1999/02/22-rdf-syntax-ns#>
prefix owl: <http://www.w3.org/2002/07/owl#>
prefix obo: <http://purl.obolibrary.org/obo/>
SELECT distinct ?label ?label2
From <http://purl.obolibrary.org/obo/merged/ODNAE>
WHERE
{ ?s rdfs:subClassOf ?s1 .
  ?s rdfs:label ?label .
  ?s1 owl:onProperty obo:RO_0000087; owl:someValuesFrom ?s2 .
  ?s2 rdfs:subClassOf <http://purl.obolibrary.org/obo/ODNAE_0000002>  option (transitive).
  ?s2 rdfs:label ?label2 .
  FILTER REGEX(str(?label2), "Agonist")
}
```

# results in 17 drugs and agonist pair: (run on http://sparql.hegroup.org/sparql/, at 10:28 CST 4/30/2015 by Asiyah Yu Lin)

label label2
cevimeline Cholinergic Muscarinic Agonists [MoA]
Pergolide Oral Tablet Dopamine Agonists [MoA]
……
Megestrol Oral Suspension Progestational Hormone Receptor Agonists [MoA]

**# SPARQL 2.4**. drugs link to antagonist

```
prefix rdf: <http://www.w3.org/1999/02/22-rdf-syntax-ns#>
prefix owl: <http://www.w3.org/2002/07/owl#>
```

```
prefix obo: <http://purl.obolibrary.org/obo/>
SELECT distinct ?s ?label
From <http://purl.obolibrary.org/obo/merged/ODNAE>
WHERE
{ ?s rdfs:subClassOf ?s1 .
  ?s rdfs:label ?label .
  ?s1 owl:onProperty obo:RO_0000087; owl:someValuesFrom ?s2 .
  ?s2 rdfs:subClassOf <http://purl.obolibrary.org/obo/ODNAE_0000002>  option (transitive).
  ?s2 rdfs:label ?label2 .
  FILTER REGEX(str(?label2), "Antagonist")
}
```

# results in total 16 drugs: (run on http://sparql.hegroup.org/sparql/, at 10:00 CST 4/30/2015 by Asiyah Yu Lin)

| s | label |
|---|---|
| http://purl.obolibrary.org/obo/DRON_00022309 | Losartan Oral Tablet |
| http://purl.obolibrary.org/obo/DRON_00020523 | Amiodarone Oral Tablet |
| …… | |
| http://purl.obolibrary.org/obo/DRON_00020960 | bicalutamide Oral Tablet |

**# SPARQL 2.5**.The antagnoist that links to ODNAE drugs

```
prefix rdf: <http://www.w3.org/1999/02/22-rdf-syntax-ns#>
prefix owl: <http://www.w3.org/2002/07/owl#>
prefix obo: <http://purl.obolibrary.org/obo/>
SELECT distinct ?s2 ?label2
From <http://purl.obolibrary.org/obo/merged/ODNAE>
WHERE
{ ?s rdfs:subClassOf ?s1 .
  ?s rdfs:label ?label .
  ?s1 owl:onProperty obo:RO_0000087; owl:someValuesFrom ?s2 .
  ?s2 rdfs:subClassOf <http://purl.obolibrary.org/obo/ODNAE_0000002>  option (transitive).
  ?s2 rdfs:label ?label2 .
  FILTER REGEX(str(?label2), "Antagonist")
}
```

# results in total 17 antagnoist: (run on http://sparql.hegroup.org/sparql/, at 10:01 CST 4/30/2015 by Asiyah Yu Lin)

| s2 | label2 |
|---|---|
| http://evs.nci.nih.gov/ftp1/NDF-RT/NDF-RT.owl#N0000000070 | Angiotensin 2 Receptor Antagonists [MoA] |
| http://evs.nci.nih.gov/ftp1/NDF-RT/NDF-RT.owl#N0000000069 | Calcium Channel Antagonists [MoA] |
| …… | |
| http://evs.nci.nih.gov/ftp1/NDF-RT/NDF-RT.owl#N0000000243 | Androgen Receptor Antagonists [MoA] |

**# SPARQL 2.6**. Antagonist and drugs pair

```
prefix rdf: <http://www.w3.org/1999/02/22-rdf-syntax-ns#>
prefix owl: <http://www.w3.org/2002/07/owl#>
prefix obo: <http://purl.obolibrary.org/obo/>
SELECT distinct ?label ?label2
From <http://purl.obolibrary.org/obo/merged/ODNAE>
WHERE
{ ?s rdfs:subClassOf ?s1 .
  ?s rdfs:label ?label .
  ?s1 owl:onProperty obo:RO_0000087; owl:someValuesFrom ?s2 .
  ?s2 rdfs:subClassOf <http://purl.obolibrary.org/obo/ODNAE_0000002>  option (transitive).
  ?s2 rdfs:label ?label2 .
  FILTER REGEX(str(?label2), "Antagonist")
}
```

# results in 21 drugs and agonist pair: (run on http://sparql.hegroup.org/sparql/, at 10:28 CST 4/30/2015 by Asiyah Yu Lin)

| label | label2 |
|---|---|
| Losartan Oral Tablet | Angiotensin 2 Receptor Antagonists [MoA] |
| Amiodarone Oral Tablet | Calcium Channel Antagonists [MoA] |
| …… | |
| bicalutamide Oral Tablet | Androgen Receptor Antagonists [MoA] |

**# SPARQL 2.7**. drug link to both a kind of agonist and antagonist

```
prefix rdf: <http://www.w3.org/1999/02/22-rdf-syntax-ns#>
prefix owl: <http://www.w3.org/2002/07/owl#>
prefix obo: <http://purl.obolibrary.org/obo/>
SELECT distinct ?s ?label ?label2 ?label4
From <http://purl.obolibrary.org/obo/merged/ODNAE>
WHERE
{ ?s rdfs:subClassOf ?s1 .
  ?s rdfs:label ?label .

  ?s1 owl:onProperty obo:RO_0000087; owl:someValuesFrom ?s2 .
  ?s2 rdfs:subClassOf <http://purl.obolibrary.org/obo/ODNAE_0000002>  option (transitive).
  ?s2 rdfs:label ?label2 .
  FILTER REGEX(str(?label2), "Agonist")

  ?s rdfs:subClassOf ?s3 .
  ?s3 owl:onProperty obo:RO_0000087; owl:someValuesFrom ?s4 .
  ?s4 rdfs:subClassOf <http://purl.obolibrary.org/obo/ODNAE_0000002>  option (transitive).
  ?s4 rdfs:label ?label4 .
  FILTER REGEX(str(?label4), "Antagonist")
}
```

# results in 1 drug : (run on http://sparql.hegroup.org/sparql/, at 12:10 CST 4/30/2015 by Asiyah Yu Lin)
--

**# SPARQL 2.8**. query drugs that have a specific role, e.g., Serotonin Agonists [MoA] (N0000000256).
# Note: This is the SPARQL script for Figure 6.
# Goal: Find drugs that has role (RO_0000087) of Serotonin Agonists [MoA] (N0000000256) from ODNAE

```
prefix owl: <http://www.w3.org/2002/07/owl#>
prefix obo: <http://purl.obolibrary.org/obo/>
prefix ndfrt: <http://evs.nci.nih.gov/ftp1/NDF-RT/NDF-RT.owl#>

SELECT distinct ?drug ?drug_label
FROM <http://purl.obolibrary.org/obo/merged/ODNAE>
WHERE {
  ?drug rdfs:subClassOf ?bnode .
  ?drug rdfs:label ?drug_label .
  ?bnode owl:onProperty obo:RO_0000087; owl:someValuesFrom ndfrt:N0000000256 .
}
```

## The Content of SPARQL File 3:

Script file name: **query_all_drug_with_MoAs**
Content:
--
**# SPARQL 3.1.** SPARQL query for searching all ODNAE drugs link to MoAs : subClassOf http://purl.obolibrary.org/obo/ODNAE_0000002 (role in cellular and molecular interactions)

```
prefix rdf: <http://www.w3.org/1999/02/22-rdf-syntax-ns#>
prefix owl: <http://www.w3.org/2002/07/owl#>
prefix obo: <http://purl.obolibrary.org/obo/>
SELECT distinct ?s ?label
From <http://purl.obolibrary.org/obo/merged/ODNAE>
WHERE
{ ?s rdfs:subClassOf ?s1 .
  ?s rdfs:label ?label .
  ?s1 owl:onProperty obo:RO_0000087; owl:someValuesFrom ?s2 .
  ?s2 rdfs:subClassOf <http://purl.obolibrary.org/obo/ODNAE_0000002>  option (transitive).
}
```

# results in total 162 drugs: (run on http://sparql.hegroup.org/sparql/, at 10:03 CST 4/30/2015 by Asiyah Yu Lin)

| s | label |
|---|---|
| http://purl.obolibrary.org/obo/DRON_00020539 | anastrozole Oral Tablet |
| http://purl.obolibrary.org/obo/DRON_00022714 | Nitrofurantoin Oral Tablet |
| …… | |
| http://purl.obolibrary.org/obo/DRON_00020960 | bicalutamide Oral Tablet |

--

## The Content of SPARQL 4:

Script file name: **query_neurotransmitters_interaction_drugs**
Content:
--
**# SPARQL 4.1.** SPARQL query for searching the ODNAE drugs annotated with Neurotransmitter Transporter Interactions [MoA] (<http://evs.nci.nih.gov/ftp1/NDF-RT/NDF-RT.owl#N0000000105>) and its subclasses.

```
prefix rdf: <http://www.w3.org/1999/02/22-rdf-syntax-ns#>
prefix owl: <http://www.w3.org/2002/07/owl#>
prefix obo: <http://purl.obolibrary.org/obo/>
SELECT distinct ?s ?label
From <http://purl.obolibrary.org/obo/merged/ODNAE>
WHERE
{ ?s rdfs:subClassOf ?s1 .
  ?s rdfs:label ?label .
  ?s1 owl:onProperty obo:RO_0000087; owl:someValuesFrom ?s2 .
  ?s2 rdfs:subClassOf <http://evs.nci.nih.gov/ftp1/NDF-RT/NDF-RT.owl#N0000000105>  option
(transitive).
}
```

# results in total 12 drugs: (run on http://sparql.hegroup.org/sparql/, at 8:56 CST 4/30/2015 by Asiyah Yu Lin)

| s | label |
|---|---|
| http://purl.obolibrary.org/obo/DRON_00026665 | Bupropion Oral Tablet |
| http://purl.obolibrary.org/obo/DRON_00020525 | Amitriptyline Oral Tablet |
| …… | |
| http://purl.obolibrary.org/obo/DRON_00027059 | Paroxetine Oral Tablet |

**# SPARQL 4.2.** # SPARQL query for searching the ODNAE drugs annotated with Dopamine Transporter Interactions [MoA] (<http://evs.nci.nih.gov/ftp1/NDF-RT/NDF-RT.owl#N0000000219>) and its subclasses.

```
prefix rdf: <http://www.w3.org/1999/02/22-rdf-syntax-ns#>
prefix owl: <http://www.w3.org/2002/07/owl#>
prefix obo: <http://purl.obolibrary.org/obo/>
SELECT distinct ?s ?label
From <http://purl.obolibrary.org/obo/merged/ODNAE>
WHERE
{ ?s rdfs:subClassOf ?s1 .
  ?s rdfs:label ?label .
  ?s1 owl:onProperty obo:RO_0000087; owl:someValuesFrom ?s2 .
```

```
  ?s2 rdfs:subClassOf <http://evs.nci.nih.gov/ftp1/NDF-RT/NDF-RT.owl#N0000000219>  option
(transitive).
}
```

# results in total 1 drug: (run on http://sparql.hegroup.org/sparql/, at 8:59 CST 4/30/2015 by Asiyah Yu Lin)

```
s           label
http://purl.obolibrary.org/obo/DRON_00026665           Bupropion Oral Tablet
```

**# SPARQL 4.3.** SPARQL query for searching the ODNAE drugs annotated with Norepinephrine Transporter Interactions [MoA] (<http://evs.nci.nih.gov/ftp1/NDF-RT/NDF-RT.owl#N0000000234>) and its subclasses.

```
prefix rdf: <http://www.w3.org/1999/02/22-rdf-syntax-ns#>
prefix owl: <http://www.w3.org/2002/07/owl#>
prefix obo: <http://purl.obolibrary.org/obo/>
SELECT distinct ?s ?label
From <http://purl.obolibrary.org/obo/merged/ODNAE>
WHERE
{ ?s rdfs:subClassOf ?s1 .
  ?s rdfs:label ?label .
  ?s1 owl:onProperty obo:RO_0000087; owl:someValuesFrom ?s2 .
  ?s2 rdfs:subClassOf <http://evs.nci.nih.gov/ftp1/NDF-RT/NDF-RT.owl#N0000000234>  option
(transitive).
}
```

# results in total 10 drug: (run on http://sparql.hegroup.org/sparql/, at 8:59 CST 4/30/2015 by Asiyah Yu Lin)

```
s           label
http://purl.obolibrary.org/obo/DRON_00020525           Amitriptyline Oral Tablet
http://purl.obolibrary.org/obo/DRON_00020620           Imipramine Oral Tablet
……
http://purl.obolibrary.org/obo/DRON_00026665           Bupropion Oral Tablet
```

**# SPARQL 4.4.** SPARQL query for searching the ODNAE drugs annotated with Serotonin Transporter Interactions [MoA] (<http://evs.nci.nih.gov/ftp1/NDF-RT/NDF-RT.owl#N0000000167>) and its subclasses.

```
prefix rdf: <http://www.w3.org/1999/02/22-rdf-syntax-ns#>
prefix owl: <http://www.w3.org/2002/07/owl#>
prefix obo: <http://purl.obolibrary.org/obo/>
SELECT distinct ?s ?label
From <http://purl.obolibrary.org/obo/merged/ODNAE>
WHERE
{ ?s rdfs:subClassOf ?s1 .
```

?s rdfs:label ?label .
?s1 owl:onProperty obo:RO_0000087; owl:someValuesFrom ?s2 .
?s2 rdfs:subClassOf <http://evs.nci.nih.gov/ftp1/NDF-RT/NDF-RT.owl#N0000000167> option (transitive).
}

# results in total 11 drug: (run on http://sparql.hegroup.org/sparql/, at 9:05 CST 4/30/2015 by Asiyah Yu Lin)

s label
http://purl.obolibrary.org/obo/DRON_00020525 Amitriptyline Oral Tablet
http://purl.obolibrary.org/obo/DRON_00020620 Imipramine Oral Tablet
……
http://purl.obolibrary.org/obo/DRON_00027059 Paroxetine Oral Tablet

--

## The Content of SPARQL File 5:

Script file name: **query_chemicals_and_drugs**
Content:
--
**# SPARQL 5.1.** identify the drugs having proper part of chemical elements
# Note: http://www.obofoundry.org/ro/ro.owl#has_proper_part

prefix rdf: <http://www.w3.org/1999/02/22-rdf-syntax-ns#>
prefix owl: <http://www.w3.org/2002/07/owl#>
prefix obo: <http://purl.obolibrary.org/obo/>

SELECT ?d ?label1 ?c ?label2
FROM <http://purl.obolibrary.org/obo/merged/ODNAE>
WHERE {
?d rdfs:subClassOf ?s1 .
?d rdfs:label ?label1 .
?s1 owl:onProperty <http://www.obofoundry.org/ro/ro.owl#has_proper_part>; owl:someValuesFrom ?c .
?c rdfs:label ?label2 .
}

#Results:
#count (distinct ?d) → 213

Example:

| d | label1 | c | label2 |
|---|---|---|---|
| http://purl.obolibrary.org/obo/DRON_00020336 | Triamcinolone Oral Tablet | http://purl.obolibrary.org/obo/CHEBI_9667 | Triamcinolone |
| http://purl.obolibrary.org/obo/DRON_00020337 | Dexamethasone Ophthalmic Ointment | http://purl.obolibrary.org/obo/CHEBI_41879 | dexamethasone |

--

**# SPARQL 5.2.** identify all ancestor CHEBI terms of CHEBI_9667 (Triamcinolone)

```
prefix rdf: <http://www.w3.org/1999/02/22-rdf-syntax-ns#>
prefix owl: <http://www.w3.org/2002/07/owl#>
prefix obo: <http://purl.obolibrary.org/obo/>

SELECT distinct ?label_9667 ?s ?label
FROM <http://purl.obolibrary.org/obo/merged/ODNAE>
WHERE {
<http://purl.obolibrary.org/obo/CHEBI_9667> rdfs:label ?label_9667 .
<http://purl.obolibrary.org/obo/CHEBI_9667> rdfs:subClassOf ?s option (transitive) .
   ?s rdfs:label ?label .
FILTER REGEX(str(?s), "CHEBI") .
}
```

# result
13

| label_9667 | s | label |
|---|---|---|
| Triamcinolone | http://purl.obolibrary.org/obo/CHEBI_50830 | fluorinated steroid |
| Triamcinolone | http://purl.obolibrary.org/obo/CHEBI_35341 | steroid |

…

**# SPARQL 5.3**: get drugs for all child levels of a parent CHEBI term:

# CHEBI_22507: glycoside antibiotic

```
prefix rdf: <http://www.w3.org/1999/02/22-rdf-syntax-ns#>
prefix owl: <http://www.w3.org/2002/07/owl#>
prefix obo: <http://purl.obolibrary.org/obo/>

SELECT distinct ?s ?d ?d2 ?label2
FROM <http://purl.obolibrary.org/obo/merged/ODNAE>
WHERE {
   ?s rdfs:subClassOf <http://purl.obolibrary.org/obo/CHEBI_22507> option (transitive) .
?d owl:onProperty <http://www.obofoundry.org/ro/ro.owl#has_proper_part>; owl:someValuesFrom ?s .

   ?d2 rdfs:subClassOf ?d .
   ?d2 rdfs:label ?label2 .
}
```

# results: 2

| s | d | d2 | label2 |
|---|---|---|---|
| http://purl.obolibrary.org/obo/CHEBI_17833 | nodeID://b339944739 | http://purl.obolibrary.org/obo/DRON_00022009 | Gentamicin Sulfate (USP) Injectable Solution |
| http://purl.obolibrary.org/obo/CHEBI_17076 | nodeID://b339944979 | http://purl.obolibrary.org/obo/DRON_00030017 | Streptomycin Oral Capsule |

--

**# SPARQL 5.4.** Goal: get drugs for all levels of chebi terms --> #2 cleanup

```
# CHEBI_33582: carbon group molecular entities

prefix rdf: <http://www.w3.org/1999/02/22-rdf-syntax-ns#>
prefix owl: <http://www.w3.org/2002/07/owl#>
prefix obo: <http://purl.obolibrary.org/obo/>

SELECT distinct
<http://purl.obolibrary.org/obo/CHEBI_33582> ?label_targetchebi ?chebi_for_drug ?drug ?labeldrug
FROM <http://purl.obolibrary.org/obo/merged/ODNAE>
WHERE {
   <http://purl.obolibrary.org/obo/CHEBI_33582> rdfs:label ?label_targetchebi .
   ?chebi_for_drug rdfs:subClassOf <http://purl.obolibrary.org/obo/CHEBI_33582> option (transitive) .
?d owl:onProperty <http://www.obofoundry.org/ro/ro.owl#has_proper_part>;
owl:someValuesFrom ?chebi_for_drug .

   ?drug rdfs:subClassOf ?d .
   ?drug rdfs:label ?labeldrug .
}
```

#part of results:

| callret-0 | label_targetchebi | chebi_for_drug | drug | labeldrug |
|---|---|---|---|---|
| http://purl.obolibrary.org/obo/CHEBI_33582 | carbon group molecular entity | http://purl.obolibrary.org/obo/CHEBI_63581 | http://purl.obolibrary.org/obo/DRON_00023447 | Stavudine Oral Capsule |
| http://purl.obolibrary.org/obo/CHEBI_33582 | carbon group molecular entity | http://purl.obolibrary.org/obo/CHEBI_127780 | http://purl.obolibrary.org/obo/DRON_00025587 | Foscarnet Injectable Solution |
| http://purl.obolibrary.org/obo/CHEBI_33582 | carbon group molecular entity | http://purl.obolibrary.org/obo/CHEBI_44915 | http://purl.obolibrary.org/obo/DRON_00026059 | Propofol Injectable Suspension |

Note: removed this line:  ?chebi_for_drug rdfs:label ?label_chebi .
Why: because the label_chebi can have duplicate names for the same chebi.

## The Content of SPARQL 6:

Script file name: **link_drugs_Chebi_AEs**
Content:
--
**# SPARQL 6.1**: This is to find drugs vs AEs that are associated with these drugs.

```
# BFO_0000057: has_participant

prefix rdf: <http://www.w3.org/1999/02/22-rdf-syntax-ns#>
prefix owl: <http://www.w3.org/2002/07/owl#>
prefix obo: <http://purl.obolibrary.org/obo/>
```

```
SELECT  ?p_dae ?labelp ?dae ?label1 ?drug ?labeldrug ?s1
FROM <http://purl.obolibrary.org/obo/merged/ODNAE>
WHERE {
   ?dae rdfs:subClassOf ?s1 .

   ?dae rdfs:subClassOf ?p_dae .
?p_dae a  owl:Class .
?p_dae rdfs:label ?labelp .

   ?dae rdfs:label ?label1 .
   ?s1 owl:onProperty obo:BFO_0000057; owl:someValuesFrom ?drug .
   ?drug rdfs:label ?labeldrug .

FILTER REGEX(str(?p_dae), "OAE") .
}
```

#part of results:

| p_dae | labelp | dae | label1 | drug | labeldrug | s1 |
|---|---|---|---|---|---|---|
| http://purl.obolibrary.org/obo/OAE_0000337 | peripheral neuropathy AE | http://purl.obolibrary.org/obo/ODNAE_0000023 | ciprofloxacin hydrochloride (Ciloxan, Cipro, Cetraxal)-associated peripheral neuropathy AE | http://purl.obolibrary.org/obo/DRON_00021347 | Ciprofloxacin Oral Tablet | nodeID://b339945066 |
| http://purl.obolibrary.org/obo/OAE_0001215 | neurotoxicity AE | http://purl.obolibrary.org/obo/ODNAE_0000024 | cefepime (Maxipime)-associated neurotoxicity AE | http://purl.obolibrary.org/obo/DRON_00024830 | cefepime Injectable Solution | nodeID://b339945067 |
| http://purl.obolibrary.org/obo/OAE_0000418 | neuropathy AE | http://purl.obolibrary.org/obo/ODNAE_0000025 | penicillin G benzathine (Bicillin L-A)-associated neuropathy AE | http://purl.obolibrary.org/obo/DRON_00034441 | penicillin G benzathine 115 MG/ML Oral Solution | nodeID://b339945068 |

--

**# SPARQL 6.2**: This is to find AE and related drugs for the AE.

#Note: an AE (e.g., ciprofloxacin hydrochloride (Ciloxan, Cipro, Cetraxal)-associated peripheral neuropathy AE or ODNAE_0000023) may have two superclasses, e.g., peripheral neuropathy AE, and drug-associated peripheral neuropathy adverse event. And this AE ‘has participant’ some ‘Ciprofloxacin Oral Tablet’.

```
prefix rdf: <http://www.w3.org/1999/02/22-rdf-syntax-ns#>
prefix owl: <http://www.w3.org/2002/07/owl#>
prefix obo: <http://purl.obolibrary.org/obo/>

SELECT  ?p ?labelp ?d ?label1 ?c ?label2 ?s1
FROM <http://purl.obolibrary.org/obo/merged/ODNAE>
WHERE {
   ?d rdfs:subClassOf ?s1 .
   ?d rdfs:subClassOf ?p .
```

```
   ?p a  owl:Class .
   ?p rdfs:label ?labelp .

   ?d rdfs:label ?label1 .
   ?s1 owl:onProperty obo:BFO_0000057; owl:someValuesFrom ?c .
   ?c rdfs:label ?label2 .

FILTER REGEX(str(?p), "OAE") .
}
```

#part of results:

| p | labelp | d | label1 | c | label2 | s1 |
|---|---|---|---|---|---|---|
| http://purl.obolibrary.org/obo/OAE_0000337 | peripheral neuropathy AE | http://purl.obolibrary.org/obo/ODNAE_0000023 | ciprofloxacin hydrochloride (Ciloxan, Cipro, Cetraxal)-associated peripheral neuropathy AE | http://purl.obolibrary.org/obo/DRON_00021347 | Ciprofloxacin Oral Tablet | nodeID://b339945066 |
| http://purl.obolibrary.org/obo/OAE_0001215 | neurotoxicity AE | http://purl.obolibrary.org/obo/ODNAE_0000024 | cefepime (Maxipime)-associated neurotoxicity AE | http://purl.obolibrary.org/obo/DRON_00024830 | cefepime Injectable Solution | nodeID://b339945067 |
| http://purl.obolibrary.org/obo/OAE_0000418 | neuropathy AE | http://purl.obolibrary.org/obo/ODNAE_0000025 | penicillin G benzathine (Bicillin L-A)-associated neuropathy AE | http://purl.obolibrary.org/obo/DRON_00034441 | penicillin G benzathine 115 MG/ML Oral Solution | nodeID://b339945068 |

**# SPARQL 6.3:** This is to identify chebi chemical terms for a specific AE.
#Note: Basicaly, this is to merge the above two scripts to map Chebi terms directly AE terms.
#Note:  This is the script used to generate data for heatmap.

```
prefix rdf: <http://www.w3.org/1999/02/22-rdf-syntax-ns#>
prefix owl: <http://www.w3.org/2002/07/owl#>
prefix obo: <http://purl.obolibrary.org/obo/>

SELECT distinct
<http://purl.obolibrary.org/obo/CHEBI_33582> ?label_targetchebi ?chebi_for_drug ?drug ?labeldrug ?p
?labelp
FROM <http://purl.obolibrary.org/obo/merged/ODNAE>
WHERE {
   <http://purl.obolibrary.org/obo/CHEBI_33582> rdfs:label ?label_targetchebi .
   ?chebi_for_drug rdfs:subClassOf <http://purl.obolibrary.org/obo/CHEBI_33582> option (transitive) .
   ?d owl:onProperty <http://www.obofoundry.org/ro/ro.owl#has_proper_part>;
owl:someValuesFrom ?chebi_for_drug .

   ?drug rdfs:subClassOf ?d .
   ?drug rdfs:label ?labeldrug .

   ?dae rdfs:subClassOf ?s1 .
?dae rdfs:subClassOf ?p .
?p a owl:Class .
?p rdfs:label ?labelp .
```

?s1 owl:onProperty obo:BFO_0000057; owl:someValuesFrom ?drug .

FILTER REGEX(str(?p), "OAE") .

}

#part of results:

| callret-0 | label_target chebi | chebi_for_drug | drug | labeldrug | p | labelp |
|---|---|---|---|---|---|---|
| http://purl.obolibrary.org/obo/CHEBI_33582 | carbon group molecular entity | http://purl.obolibrary.org/obo/CHEBI_63581 | http://purl.obolibrary.org/obo/DRON_00023447 | Stavudine Oral Capsule | http://purl.obolibrary.org/obo/OAE_0000337 | peripheral neuropathy AE |
| http://purl.obolibrary.org/obo/CHEBI_33582 | carbon group molecular entity | http://purl.obolibrary.org/obo/CHEBI_127780 | http://purl.obolibrary.org/obo/DRON_00025587 | Foscarnet Injectable Solution | http://purl.obolibrary.org/obo/OAE_0000418 | neuropathy AE |
| http://purl.obolibrary.org/obo/CHEBI_33582 | carbon group molecular entity | http://purl.obolibrary.org/obo/CHEBI_44915 | http://purl.obolibrary.org/obo/DRON_00026059 | Propofol Injectable Suspension | http://purl.obolibrary.org/obo/OAE_0000418 | neuropathy AE |

**Notes:**

- Some drugs are not associated with AEs. For example, DRON_00022253 -- Levodopa Oral Tablet are not associated with any AEs.
- Some drugs may have two AEs, for example, DRON_00022291 - lomefloxacin Oral Tablet
